# What out-of-hours antibiotic prescribing practices are contributing to antibiotic resistance: a literature review

**DOI:** 10.29045/14784726.2020.12.4.4.25

**Published:** 2020-03-01

**Authors:** Jasmine Hart, Peter Phillips

**Affiliations:** South Western Ambulance Service NHS Foundation Trust; Bournemouth University

**Keywords:** after-hours care, antibiotic, drug resistance

## Abstract

**Background:**

Overuse of antibiotics and inappropriate prescribing has resulted in a rapid increase in the rate of antibiotic resistance, with poorer patient outcomes and increased health costs. In the out-of-hours setting, a high proportion of antibiotics are prescribed and practices need to improve to reduce antibiotic resistance.

**Purpose:**

To identify antibiotic prescribing practices in European out-of-hours primary care services that are contributing to antibiotic resistance.

**Design:**

The review was conducted in alignment with the PRISMA statement (Moher, Liberati, Tetzlaff, Altman, & PRISMA Group, 2009).

**Methods:**

A literature search was performed using MySearch to identify European literature. The search was focused on antibiotic/antimicrobial prescribing in an out-of-hours environment, and any reports that described factors correlating with the nature of prescribing practices were examined.

**Results:**

The literature search located 91 articles, out of which seven met the inclusion criteria. Two articles described clinicians’ experiences in antibiotic prescribing in out-of-hours, two compared in-office and after-hours prescribing, two described prescribing patterns in out-of-hours and one examined prescribing in children. Four main themes were identified: antibiotics prescribed and conditions associated with prescribing; consultation time; the day of consultation; and parental opinion.

**Conclusion:**

Overprescribing to self-limiting conditions, prescribing of broad-spectrum antibiotics, time constraints, safeguarding issues and poor communication are all contributing to inappropriate antibiotic prescribing. Further research is needed relating to whether clinicians are adhering to antibiotic guidelines and to explore patients’ experiences and expectations from the out-of-hours practitioners with respect to antibiotic prescribing.

## Introduction

Antimicrobial resistance is considered a serious threat to global public health (World Health Organization, 2017), with an increasing number of resistant microbial strains being reported each year (Kumarasamy et al., 2010). The unnecessary prescribing of antibiotics vastly contributes to the spread of resistance (Costelloe, Metcalfe, Lovering, Mant, & Hay, 2010) and is associated with poorer patient outcomes and increased healthcare costs (Cosgrove, 2006). Antibiotics are medicines used to prevent and treat bacterial infections, while the term antimicrobial encompasses other microbes as well, such as parasites, viruses and fungi (World Health Organization, 2019). In the United Kingdom, at least 20% of all antibiotic treatment in primary care is inappropriately prescribed (Public Health England, 2016). The out-of-hours (OOH) system has been identified by Public Health England (2016) as accounting for 4.7% of prescribing in the community setting, which accounted for approximately 5,197,311 prescriptions in the United Kingdom in 2017 (Prescribing and Medicine Team, 2018). By understanding the factors influencing prescribing in OOH, it may enable appropriate interventions to promote optimal antibiotic therapy in this environment. Due to a change in legislation that permits paramedics to prescribe (once the appropriate qualifications have been obtained), antimicrobial stewardship will be the responsibility of these clinicians too. This literature review aims to identify what OOH prescribing practices may be contributing to antimicrobial resistance.

## Methods

### Design

A literature review was conducted in accordance with the PRISMA (Preferred Reporting Items for Systematic Reviews and Meta-Analyses) statement (Moher, Liberati, Tetzlaff, Altman, & PRISMA Group, 2009; Supplementary 1). The Bournemouth university database, MySearch, was used to conduct the review. This database combines results from CINAHL Complete, Cochrane Library, EBSCO, MEDLINE, PubMed and the Trip database. Both authors conducted the search independently, including conducting quality appraisal, and then compared results to decide which studies should be included in the final review. Disagreements were discussed and a decision reached collaboratively.

### Search methods and outcomes

A scoping review was conducted on 15 January 2017, first to identify specific terminology within abstracts that would help to form the search terms for this literature review. Based on this work, the search terms chosen for the review were ‘antibiotic prescribing’ and ‘antimicrobial prescribing’, which identified 13,764 articles, and ‘out-of-hours’, ‘pre-hospital’ and ‘after-hours care’, which identified 148,453 articles. By combining both sets of search terms, a total of 91 articles were identified.

**Figure 1:**
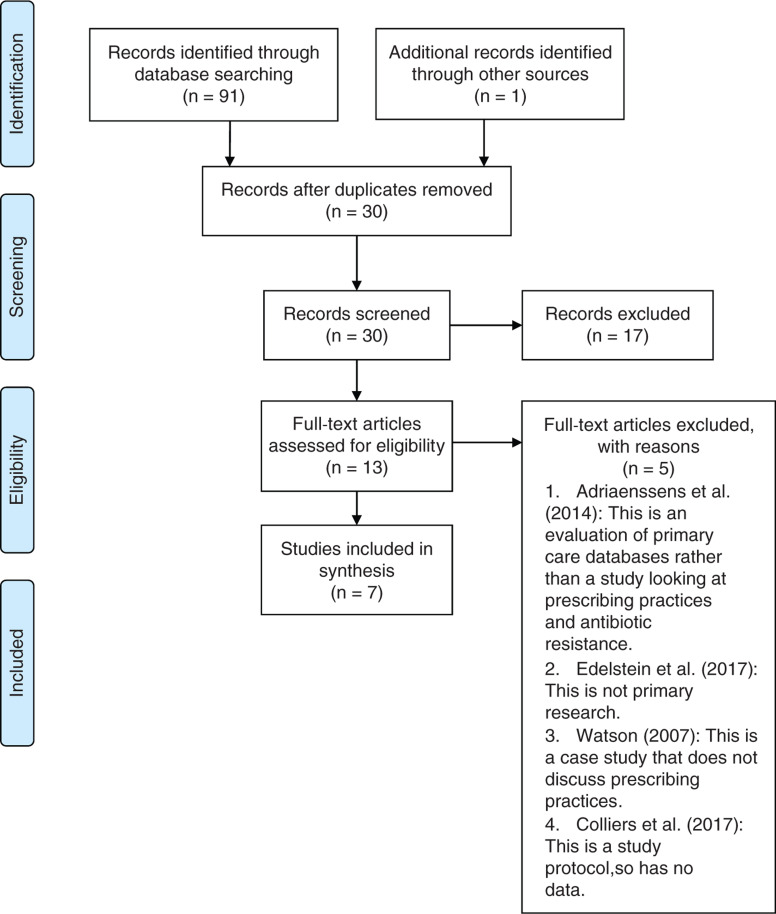
Figure 1. PRISMA flow diagram of search results (Moher et al., 2009).

A search limit of 2004 was initially considered, as this is when the NHS created an integrated OOH system (National Audit Office, 2014), but as European studies were included, no date limits were applied. After applying search limits of the English language and peer review articles, 71 articles remained; removing duplicates further reduced this number to 29. Seventeen articles were removed due to the title or abstract not answering the research question. After reading, a further six were discounted for not answering the research question. This left a total of seven articles: the six original papers plus an additional paper that was identified in the Oxford academic database at a later date (Figure 1). No further papers were identified from reference lists.

### Inclusion and exclusion criteria

Articles were included if they focused on antibiotic prescribing practices in an OOH environment, were peer-reviewed journal articles, in the English language, about European studies and had been published. Exclusion criteria included trials in a hospital environment, not in the English language and studies not based in Europe.

### Quality appraisal

Quality assessment was performed using relevant Critical Appraisal Skills Programme (CASP) tools (Critical Appraisal Skills Programme, 2018), depending on the methodology of the paper. CASP was chosen to critique the papers as both quantitative and qualitative papers were identified. For each question on the CASP checklist, one point was awarded for each ‘yes’ response. This total was divided by the number of questions in the checklist, with a score of 60% set as the threshold for sufficient quality for the paper to be included in the review.

## Results

### Study design

Seven articles met the inclusion criteria and were deemed suitable for further review. Two papers were qualitative, three were cohort studies, one was a case-control study and one a secondary analysis of a randomised control trial (Table 1). All seven articles received a score of 60% or more on quality assessment; nil papers were discounted.

**Table 1.** Data extraction table.

Author (year)	Country	Study size	Participants, patients in quantitative studies and healthcare professionals in qualitative studies	Design	Outcomes	Interventions	CASP score	Strengths	Limitations
Lindberg, Gjelstad, Foshaug, & Høye (2017)	Norway	6757	Children and adults of both sexes	Quantitative cohort study	High proportion of Penicillin V prescribing at OOH compared to GP practices. Seeing more patients and having a shorter consultation time is associated with higher prescription rates. Broad spectrum antibiotics more likely to be prescribed in the elderly. Tonsillitis and sinusitis have the highest prediction values for antibiotics.	Antibiotic prescribing	10/11 90.9% Excellent	Doctors were not aware of plans for a research project at the time of consultation, so there is a limited risk of observer bias. Large study size. Rate of doctors agreeing to participate was high.	Doctors unaware of research plan, which could lead to a lack of accuracy in the diagnosis. Not identifying patients who were at the OOH due to antibiotic treatment failure from the previous consultation.
Huibers, Moth, Christensen, & Vedsted (2014)	Denmark	96,916	Children and adults of both sexes	Quantitative cohort study	Prescribing proportion highest for patients aged 0–4 years and lowest for adults aged 60+ years. Antibiotics more likely to be prescribed at weekend than weekdays. Most frequently prescribed antibiotics are beta-lactamase sensitive beta-lactams, broad spectrum beta-lactams and antibacterial eye drops.	Antibiotic prescribing	10/11 90.9% Excellent	Automatic electronic data collection ensured complete and valid data with limited risk of information or selection bias. Large study size. Over a 12-month period, so accounting for seasonal variations.	The data did not allow to review the indications for antibiotic prescriptions or measure guideline adherence.
Debets, Verheij, & van der Velden (2017)	Netherlands	6,434,640	Children and adults of both sexes	Quantitative case control study	Prescribing rates for sinusitis, tonsillitis and cystitis higher in OOH. Higher prescribing proportion of amoxicillin in OOH. OOH prescribing quality was lower.	Antibiotic prescribing compared in OOH and in-hour practices	7/9 77.7% Good	All office hours and OOH contacts from one complete year were analysed, ensuring sustainability. GPs were not aware of the analysis of their prescribing behaviours. Information on the types of prescribers present.	The analysis of OOH was done 1–2 years after the data collection. Each setting uses a different online system and when specific information was not noted, it was regarded as absent, which could lead to incorrect data.
Hayward, Fisher, Spence, & Lasserson (2016)	England	496,931	Children and adults of both sexes	Quantitative cohort study	Most antibiotics were issued on a Saturday daytime. Most commonly prescribed antibiotics were penicillins. Patient contact with OOH service reduced over four years, but antibiotic prescription rose during this period. Younger age, female sex and lower deprivation score were all interdependently associated with a higher prescription rate.	Antibiotic prescribing in OOH primary care	11/11 100% Excellent	A large sample size. Robustness of the dataset used. Comparison with other literature. Studied data from a 4-year period.	Not being able to analyse the type of consultation from the electronic records. Not being able to distinguish the ‘delayed’ antibiotic prescriptions. This could change the overall trend. Not able to compare the proportion of broad-spectrum antibiotics in each group.
de Bont et al. (2015)	Netherlands	37	GPs	Qualitative study	Decision influenced by parental opinions and whether or not the child was prescribed antibiotics previously. Overprescribing due to lack of long-term relationship with patient and time constraints. Diagnostic uncertainty led to prescribing. Patients more satisfied when prescribed antibiotics.	Experiences of GPs regarding childhood fever	9/10 90% Excellent	Heterogeneous sample which improves transferability of the results. Qualitative design made it possible to research the complexity of the topic. Used an independent moderator asking open-ended questions to reduce influence of researchers’ opinions.	Focused on more than one topic. High probability of selection bias. Focus groups could lead to opinions and experiences of participants being altered.
Rebnord, Sandvik, Mjelle, & Hunskaar (2017)	Norway	397	Children aged 0–6 years, both sexes	Quantitative, secondary analysis of a randomised controlled study	CRP > 20, use of paracetamol and signs on ear examination were the main predictors of antibiotic prescribing. Strong association between parent opinion and treatment.	CRP testing	6/9 66.6% Good	Large number of participants. Small age group so results are more specific.	No details on the previous experience of the OOH doctors. Observational study of a randomised study. Doctors were informed about the study.
Williams et al. (2018)	United Kingdom	30	GPs and NPs	Qualitative study	NPs more likely to adhere to protocols. Higher prescribing rates at weekends as additional safety netting. Favoured delayed prescribing. Harder to deny antibiotics to patients who have received them previously. More pressure to prescribe due to not having previous ePRs and not being able to follow up. Consultation time and pressure influenced antibiotic prescribing. A patient’s inability to re-access OOH, should they need to, suggested an increased likelihood of antibiotics being prescribed.	Experiences of GPs and NPs of prescribing antibiotics for respiratory tract infections in OOH.	8/10 80% Excellent	Findings resonate with other published literature. Wide variation of participants from different backgrounds and work locations. Interviews were audio recorded.	Risk of bias due to purposive sampling. Small response rate.

Note: CASP = Critical Appraisal Skills Programme; CRP = C-Reactive Protein; ePR = Electronic Patient Record; NP = Nurse Practitioner; OOH = Out-of-Hours. Marks were calculated to give a percentage. A percentage over 60% was included and deemed to be good quality evidence; anything below this was not included in the results. A mark over 80% was deemed to be excellent quality evidence.

### Demographic and location

The location of the studies varied throughout Europe: two studies were based in the United Kingdom, two in the Netherlands, two in Norway and one in Denmark. Three of the papers included healthcare professionals as their participants while four studies used patients as their target groups. Four of the papers had both child and adult patient participation groups of both sexes. Female patients between the ages of 18 and 60 were most likely to receive a prescription. Williams et al. (2018) and de Bont et al. (2015) interviewed staff regarding their prescribing practices relating respectively to children aged 0–12 of both sexes and for all ages and sexes of the patient. Rebnord, Sandvik, Mjelle and Hunskaar (2017) performed a secondary analysis of a randomised control trial that focused solely on children aged 0–6 years old.

Williams et al. (2018) did not identify the age or sex of the practitioners, but de Bont et al. (2015) reported that staff had a mean age of 47 years and the majority of participants were male. Large sample sizes were present in all the quantitative studies, the largest being the study by Debets, Verheij and van der Velden (2017), which analysed 6,434,640 antibiotic courses dispensed by community pharmacies and the smallest being the study by Rebnord et al. (2017), which had 397 child participants. All other quantitative studies had sample sizes that ranged between 6757 and 496,931. Sample sizes in the qualitative papers that interviewed staff were between 30 and 37.

### Strengths and limitations

Overall, the sample sizes of the included studies seemed adequate; even the qualitative papers had appropriate numbers of participants for their methodology. For example, Williams et al. (2018) recruited 30 participants. In the studies where both adults and children participated, the studies were retrospective, and the GPs were unaware that a research study was taking place, which is ultimately beneficial as it reduces the chance of selection bias. The rate of doctors agreeing to participate was high, giving more valid results and reflecting clinical reality.

The qualitative articles by de Bont et al. (2015) and Williams et al. (2018) both used a semi-structured interview to guide their questions, which were audio recorded. For the interview process, de Bont et al. (2015) used focus groups, facilitated by an experienced and independent moderator, while Williams et al. (2018) interviewed over the telephone and two qualitative researchers asked the questions. Both methods come with their strengths and limitations. De Bont et al. (2015) and Williams et al. (2018) both have a risk of recall bias, as they rely on participants recounting previous experiences, which may have been remembered differently over time.

De Bont et al. (2015) and Rebnord et al. (2017) included several topics of focus other than antibiotic prescribing, which is considered a limitation in this review. Lindberg, Gjelstad, Foshaug and Høye (2017) and Hayward, Fisher, Spence and Lasserson (2016) had difficulties in certain aspects of their studies. They were not able to identify patients who were at OOH due to antibiotic treatment failure from previous consultations, nor were they able to determine whether the patients received a delayed antibiotic prescription.

## Discussion

### Antibiotics prescribed and conditions associated with antibiotic prescribing

Antibiotic consumption is contributing to the spread of antimicrobial resistance, and prudent antibiotic prescribing has been identified as an essential strategy to curb this problem (Dolk, Pouwels, Smith, Robotham, & Smieszek, 2018). Prudent prescribing includes prescribing narrow-spectrum antibiotics and avoiding unnecessary treatment (Dolk et al., 2018). In this literature review, four studies reported high use of penicillin in the OOH system. Huibers, Moth, Christensen and Vedsted (2014) found that the most frequently prescribed antibiotics were beta-lactamase sensitive penicillins (34.9% of total antibiotic prescriptions), followed by broad-spectrum penicillins (21%). Hayward et al. (2016) stated that phenoxymethylpenicillin (PcV), which is used first line solely for acute tonsillitis in the United Kingdom, was in the top five in all age groups except older adults. Amoxicillin accounted for 28.2% of all UK antibiotics prescribed, making it the most issued. Lindberg et al. (2017) noted that PcV was prescribed to 69.9% of patients receiving antibiotics for an acute respiratory tract infection (ARTI) and that conditions such as tonsillitis and sinusitis received substantial antibiotic treatment, 80.3% (odds ratio [OR], 21.11) and 75.9% (OR 12.39) respectively. Similarly, Debets et al. (2017) demonstrated that certain conditions which are generally self-limiting in healthy adults had a strong predictor value for an antibiotic prescription. Patients with cystitis received antibiotics in 93.9% of consultations, acute tonsillitis in 78% and sinusitis in 70%. The findings from Debets et al. (2017) are similar to the outcomes of Lindberg et al. (2017), increasing the validity of the results.

Multiple studies and current clinical management guidance suggest that tonsillitis, sinusitis and cystitis will resolve without treatment in healthy patients (National Institute for Health and Care Excellence, 2018). Uncomplicated cystitis in healthy non-pregnant women is due to urinary tract infection in 95% of general practice presentation, and is self-limiting, with symptoms typically resolving within a week (Baerheim, 2012). Despite this, cystitis accounts for a substantial proportion of antibiotics prescribed OOH (Debets et al., 2017). Kanji et al. (2016) performed a cross-sectional study to determine whether antibiotics were still appropriately prescribed for acute tonsillitis. They used the Centor score to determine the appropriateness of prescribing – a score of below three should not be considered for antibiotic therapy (National Institute for Health and Care Excellence, 2008). They found that antibiotics were prescribed to 196/246 patients diagnosed with acute tonsillitis with a Centor score of below three. Although this study was conducted in an emergency department in the United Kingdom, the fact that the diagnosis was made on symptoms alone suggests this finding is transferable to the OOH setting. The article being published in 2016 further supports that it reflects current up-to-date practice. There is also a limited chance of selection bias as this is a retrospective study.

It is apparent from the evidence that the frequent prescribing for self-limiting conditions such as cystitis, sinusitis and tonsillitis suggests room for improvement of rational antibiotic use. Stricter guidance to further reduce the number of broad-spectrum antibiotics prescribed needs to be put in place to tackle antimicrobial resistance effectively. Further research into whether clinicians are adhering to guidelines would be beneficial. This evidence is already apparent in the in-hours setting; however, there are currently no studies that have investigated why physicians are prescribing for self-limiting conditions in OOH.

### Consultation time

Two studies highlighted that time restrictions in the consultation lead to a higher antibiotic prescription rate. Lindberg et al. (2017) reported an increase in the number of prescriptions in time-pressured consultations. Williams et al. (2018) agreed with Lindberg et al. (2017) and described that a short consultation due to an unrealistic work volume largely influenced a clinician’s decision to give antibiotics. A study by Gjelstad et al. (2011) investigated the prescribing practices of 440 GPs in Norway to demonstrate the impact of short consultation time quantitatively. They identified that the overall number of consultations per year significantly correlated with higher rates of antibiotic prescribing. GPs in the upper quintile for their annual number of consultations (3871–11,252 consultations per year) were 1.64 times more likely to prescribe antibiotics compared to GPs in the lowest quintile (252–2113). The percentages of prescriptions for an ARTI rose from 28.1% (OR, 1.00) to 36.6% (OR, 1.64).

The reasons behind the increase in prescriptions could be down to GPs and nurse practitioners (NPs) feeling the pressure of the time constraints, not being able to discuss alternative approaches or the nature of the illness with the patient and ultimately prescribing antibiotics as a time-saving strategy. In the OOH this is intensified, with patients being more impatient and demanding antibiotics as they consider themselves more unwell (Williams et al., 2018). One solution to this could be training other practitioners such as nurses and paramedics to an independent prescribing level with the hope to reduce the workload of GPs. Additionally, an educational out-reach intervention based on national guidelines may be beneficial to reduce irresponsible prescribing.

### Day of consultation

OOH in Europe was described in most articles as services provided outside of regular working hours, typically evenings and weekends, although evening time varied by location. The evidence revealed associated implications with the day of consultation. Huibers et al. (2014) found that at weekends, antibiotics were prescribed more frequently on Saturdays. This is also acknowledged in Williams et al. (2018), and prescribing was reportedly used as an additional safety net. Hayward et al. (2016) showed that 44% of all antibiotics prescribed in the OOH setting were prescribed on Saturdays and 31.2% were prescribed on Sundays. In contrast, Huibers et al. (2014) reported that most antibiotics in their study were prescribed weekdays between 16:00 and 20:00, just after opening hours of the OOH service. The explanation for this could be that patients have difficulty accessing their GP in-hours due to limited on-the-day appointments or perhaps because most patients are working during the normal opening hours of a GP surgery. Another factor that Huibers et al. (2014) noted was that broad-spectrum antibiotics were more frequently prescribed on weekdays than at weekends. This could be related to the high influx of patients arriving when OOH initially opens, and not having time to assess patients as thoroughly as they would like, hence prescribing a broad-spectrum alternative to cover a variety of potentials. In these circumstances, delayed prescribing could be used to the clinician’s advantage. The use of delayed prescribing can limit the collection of prescriptions by patients to just 40% and is shown to be as effective as an immediate prescription in reducing complication for an ARTI (Ryves et al., 2016).

### Parental opinion

Respiratory tract infection, which is predominantly self-limiting, is the most common reason why children consult a GP (Dekker et al., 2018). De Bont et al. (2015) found that parental concern was one of the primary considerations for prescribing antibiotics, as it was closely related to parental satisfaction and avoiding discussion with parents during these time-pressured consultations. Rebnord et al. (2017) recorded parents’ opinions on whether they believed their child had a viral, bacterial or no infection before seeing the doctor. Of 116 parents who thought their child had a bacterial infection, 45 received antibiotics, and of the 101 who suspected a viral infection, only 14 were prescribed antibiotics. A systematic review by Bosley, Henshall, Appleton & Jackson (2018) found that parental perception of antibiotics was that they are ‘wonder’ drugs, which could treat any illness. The review identified that clinicians were the primary source of information for parents regarding antibiotic usage and that parents wanted to know more about appropriate prescribing but healthcare professionals used medical jargonistic language that they did not understand and did not take the time to explain the treatment. In their study, they included low-income countries that had lower levels of knowledge in comparison with western society, hence this study might not be entirely applicable to this literature review.

Overall, parents’ assessments of sickness and seriousness were significantly associated with the treatment outcomes. This may reflect that they know their child well or may suggest that clinicians often prescribe to satisfy parents. The results from Bosley et al. (2018) suggest that parents are reasonable in their expectations but need an adequate explanation using simple terms.

### Limitations

There are certain limitations to this review. ‘Grey’ literature was not accessed as it is difficult to find and beyond the scope of the literature review; therefore, it is possible that unpublished articles and non-academic articles were missed. Another limitation is that it comprised an electronic search, and despite the advancement in electronic searching, digital searching tools are not 100% comprehensive and will not identify all the literature. A further limitation is that the review was limited to literature written in the English language. Only European literature was included, hence location bias is also present in the review, as is the potential for clinical practice to vary depending on location, such as European practices varying from British practices.

## Conclusion

Overprescribing for self-limiting conditions – especially of broad-spectrum antibiotics – time constraints, safeguarding issues and poor communication are all contributing to inappropriate antibiotic prescribing. Further research is needed relating to whether clinicians are adhering to antibiotic guidelines and to explore patients’ experiences and expectations from the OOH practitioners with respect to antibiotic prescribing.

## Conflict of interest

None declared.

## Funding

None.
